# Procalcitonin Improves the Glasgow Prognostic Score for Outcome Prediction in Emergency Patients with Cancer: A Cohort Study

**DOI:** 10.1155/2015/795801

**Published:** 2015-03-15

**Authors:** Anna Christina Rast, Alexander Kutz, Susan Felder, Lukas Faessler, Deborah Steiner, Svenja Laukemann, Sebastian Haubitz, Andreas Huber, Ulrich Buergi, Antoinette Conca, Barbara Reutlinger, Beat Mueller, Mario Bargetzi, Philipp Schuetz

**Affiliations:** ^1^Medical University Department of the University of Basel, Kantonsspital Aarau, Tellstrasse, 5001 Aarau, Switzerland; ^2^Institute of Psychology, University of Bern, Muesmatt, 3012 Bern, Switzerland; ^3^University Clinic of Infectious Diseases, University Hospital Bern, Freiburgstrasse 3, 3010 Bern, Switzerland; ^4^Department of Laboratory Medicine, Kantonsspital Aarau, Tellstrasse, 5001 Aarau, Switzerland; ^5^Emergency Department, Kantonsspital Aarau, Tellstrasse, 5001 Aarau, Switzerland; ^6^Department of Clinical Nursing Science, Kantonsspital Aarau, Tellstrasse, 5001 Aarau, Switzerland; ^7^Division of Hematology & Oncology, Medical University Department of the University of Basel, Kantonsspital Aarau, Tellstrasse, 5001 Aarau, Switzerland

## Abstract

The Glasgow Prognostic Score (GPS) is useful for predicting long-term mortality in cancer patients. Our aim was to validate the GPS in ED patients with different cancer-related urgency and investigate whether biomarkers would improve its accuracy. We followed consecutive medical patients presenting with a cancer-related medical urgency to a tertiary care hospital in Switzerland. Upon admission, we measured procalcitonin (PCT), white blood cell count, urea, 25-hydroxyvitamin D, corrected calcium, C-reactive protein, and albumin and calculated the GPS. Of 341 included patients (median age 68 years, 61% males), 81 (23.8%) died within 30 days after admission. The GPS showed moderate prognostic accuracy (AUC 0.67) for mortality. Among the different biomarkers, PCT provided the highest prognostic accuracy (odds ratio 1.6 (95% confidence interval 1.3 to 1.9), *P* < 0.001, AUC 0.69) and significantly improved the GPS to a combined AUC of 0.74 (*P* = 0.007). Considering all investigated biomarkers, the AUC increased to 0.76 (*P* < 0.001). The GPS performance was significantly improved by the addition of PCT and other biomarkers for risk stratification in ED cancer patients. The benefit of early risk stratification by the GPS in combination with biomarkers from different pathways should be investigated in further interventional trials.

## 1. Background

Risk stratification in patients with solid and haematological cancer presenting to the emergency department (ED) is important. Yet, there are no well-validated risk scores for these patients in the emergency setting. In recent years, the Glasgow Prognostic Score (GPS) [1, 2], which consists of two predictors, namely, C-reactive protein (CRP) and albumin, has been found useful for predicting long-term mortality primarily in cancer patients in the outpatient or pre- and postsurgical settings [3]. A recent review analysed over 60 studies with more than 30,000 patients investigating the use of the GPS or its modified version (mGPS) (Table 1) in a variety of tumour scenarios: in unselected cohorts, in operable diseases, in radiochemotherapy, and in inoperable disease [4]. Thereby a prognostic potential was found in most settings and particularly in patients with colon cancer undergoing potentially curative therapy [5]. Yet, whether this score also allows short-term prognostication in unselected patients presenting to the ED with different cancer-related medical urgencies remains unclear.

In addition to clinical scores for risk stratification in the ED, several blood biomarkers have been put forward in ED patients for improved risk stratification [6–9]. Such markers from different physiopathological pathways may provide useful prognostic information that improves management in regard to initial site-of-care decisions (outpatient versus inpatient) and expected risks. Few reports, however, have focused on biomarkers in patients with cancer. Outside of emergency care, yet, different biomarkers have been studied for their prognostic potential in cancer patients. First, traditional inflammatory markers such as white blood cell count (WBC) and CRP have shown prognostic value in patients prior to developing a cancer or when they are already suffering from a detected cancer [10–12]. Second, procalcitonin (PCT), the precursor hormone of calcitonin, is a marker for bacterial infections with a high negative predictive value compared with blood cultures to rule out sepsis and systemic inflammation in the ED setting with also moderate prognostic accuracy [13]. Third, low levels of the 25-hydroxyvitamin D (25(OH) vitamin D) have been associated with higher risk of death from all causes, particularly if patients have a past medical history of cancer. Results of a meta-analysis therefore have suggested that 25(OH) vitamin D may be an interesting prognostic biomarker for cancer patients [14]. Fourth, kidney markers such as urea correlated with severity and outcome in different acute medical diseases including pneumonia [15] and acute heart failure [16] and may also play a role in cancer patients. Finally, calcium levels have been associated with overall and cancer-specific mortality independent of age and gender in a large cohort of 21,669 patients with cancer diagnosis [17]. Yet, the added prognostic value of biomarkers from different physiopathological pathways in unselected patients presenting with a cancer-related medical urgency to the ED remains largely unclear.

Herein, our aim was to validate the GPS in ED patients with cancer-related urgencies and investigate whether additional blood biomarkers would improve its accuracy for short-term 30-day outcome prediction.

## 2. Methods

### 2.1. Study Design and Setting

This is an observational cohort study as part of a quality control project. From February 2013 to October 2013, we included all consecutive medical patients presenting with a cancer-related medical urgency to a tertiary care hospital in Switzerland with also secondary medical care service function. As an observational quality control study, the Institutional Review Board (IRB) of the Canton of Aargau approved the study and waived the need for informed consent (EK 2012/059). The study was registered at the ClinicalTrials.gov registration website (https://www.clinicaltrials.gov/ct2/show/NCT01768494).

### 2.2. Patient Population and Management of Patients throughout the Study

We included all consecutive medical patients with a cancer-related medical urgency including (a) fever and infectious complications, (b) cancer progression or local cancer complications, and (c) worsening of general condition including radiochemotherapy related problems. Patients presenting to the surgical ward and patients <18 years of age were excluded. All procedures were carried out as part of standard patient care.

In all patients, we recorded pertinent initial vital signs (i.e., blood pressure, respiratory rate, and others) and clinical parameters (i.e., admission date, main presenting symptom, underlying type of cancer, comorbidities, type of infection, and others). In all patients, we collected leftover blood samples for later measurement of biomarkers. Clinical information including sociodemographic characteristics, comorbidities, and patient outcomes was assessed until hospital discharge using the routinely gathered information from the hospital electronic medical system used for coding of diagnosis-related groups (DRG) codes. This already available information supported the reliable assessment of baseline characteristics including demographics, comorbidities, acute medical conditions requiring the ED visit, and different patient outcomes including in-hospital mortality.

### 2.3. Definition of Groups of Diagnoses and Main Symptoms at ED Admission

Patients were grouped in different main diagnoses (infection, gastrointestinal disease, cardiovascular disease, worsening of general condition, neurological disease, and other diseases) and symptom categories (general malaise, pain, gastrointestinal symptom, neurological symptom, fever, and respiratory symptom) based on their medical charts. Thereby, the group of “neurological symptoms” included reduced consciousness, dizziness, confusion, syncope or the state shortly after, and neurological deficits.

### 2.4. Outcome

The primary endpoint of this study was all-cause mortality within 30 days and the secondary endpoint was mortality in different subgroups and according to biomarker levels. For outcome assessment, we contacted all patients by phone interview 30 days after admission to evaluate vital and functional status. In case a patient could not be reached, we contacted the family or the general practitioner to assess vital status.

### 2.5. Blood Biomarkers (including the GPS) and Their Measurement

Blood samples for later measurement of biomarkers were collected upon ED admission. The following markers were measured as part of routine care: (a) CRP ((mg/L), normal range < 3.0 mg/L, detection limit < 0.5 mg/L); (b) albumin ((g/L), normal range: 34–50 g/L); (c) WBC ((10^9^/L), normal range: 4–10 × 10^9^/L); (d) urea ((mmol/L), normal range: 2.0–7.0 mmol/L); (e) 25(OH) vitamin D ((nmol/L), normal range: 50–300 nmol/L); (f) corrected calcium ((mmol/L), normal range: 2.15–2.55 mmol/L).

In addition, we measured PCT levels post hoc with an automated rapid sensitive assay (KRYPTOR PCT; Thermo Scientific Biomarkers (formerly B·R·A·H·M·S AG), Hennigsdorf, Germany, detection limit of 0.02 *μ*g/L).

The GPS was calculated as previously recommended [1, 2]. We additionally calculated the mGPS, which has been propagated after the GPS and is based on the finding that hypoalbuminaemia without an elevated CRP was rare and on its own not associated with poor survival in patients after resection for colorectal cancer [18]. Table 1 shows the points allocated for the GPS and the mGPS.

### 2.6. Statistics

Categorical variables are expressed as percentages and counts or vice versa and continuous variables as medians (interquartile ranges, IQR, 25th–75th percentiles), unless stated otherwise. If applicable, 95% confidence intervals (95% CI) are provided. Frequency comparison was done by chi-square test and two-group comparison with nonparametric (Mann-Whitney *U*) tests. We used logistic regression models with area under the receiver operating characteristic (ROC) curve to assess associations of baseline parameters and 30-day outcomes. Thereby the area under the ROC curve (AUC) is a summary measure over criteria and cut-point choices. The AUC summary equals the probability that the underlying classifier will score a randomly drawn positive sample higher than a randomly drawn negative sample. To test whether the biomarker levels improve the GPS performance, we compared the nested logistic regression model including the GPS and biomarkers with a model limited to the GPS alone.

Statistical analyses were done with STATA 12.1 (Stata Corp, College Station, TX, USA). Testing was two-tailed; *P* < 0.05 was considered to indicate statistical significance.

## 3. Results

### 3.1. Patient Population

A total of 341 medical patients presenting with a cancer-related medical urgency were included (median age 68 years, 61.0% male gender). The main diagnoses at ED admission were infection (61.0%), neurological disease (14.7%), and cardiovascular disease (12.3%). Main symptoms were gastrointestinal (24.9%), general malaise (17.8%), fever (16.3%), and pain (14.2%). Patients had different types of solid cancers (80.9%) mainly of the gastrointestinal (25.8%) and of the respiratory tract (22.3%) as well as nonsolid cancers (17.6%) and not specified or other (1.5%). Additional baseline information is presented in Table 2.

### 3.2. Association of GPS with Outcome

Overall the GPS was significantly associated with 30-day mortality as evidenced by an odds ratio (OR) of 6.4 (95% CI 2.5 to 16.6, *P* < 0.001) at the two-point cut-off. The prognostic accuracy was moderate with an AUC of 0.67 (95% CI 0.62 to 0.72). In comparison, the mGPS had a lower discriminatory value with an AUC of 0.65 (95% CI 0.59 to 0.71), for which reason we focused on the original GPS for all analyses.

Table 3 shows detailed results of GPS performance in different predefined subgroups. The best performance concerning cancer type was found in patients with gastrointestinal (GI) cancers (AUC 0.68, 95% CI 0.59 to 0.76) and in patients presenting with predominantly neurologic symptoms (AUC 0.73, 95% CI 0.54 to 0.93). When all GI cancers were excluded, the AUC was similar (AUC 0.67, 95% CI 0.60 to 0.73) to the overall population. In addition, for the 133 patients without a main diagnosis of infection, an OR of 8.4 (95% CI 1.9 to 38.2, *P* = 0.01) at the two-point GPS cut-off was found with an AUC of 0.72 (95% CI 0.64 to 0.80) for mortality prediction.

Overall, at the one-point cut-off, the GPS had a high negative predictive value (92.5%, (95% CI 83.4% to 97.5%)) to rule out 30-day mortality (sensitivity: 93.8% (95% CI 86.2% to 98.0%)). Conversely, at the two-point cut off, the positive predictive value for mortality was low (34.0% (95% CI 27.5% to 41.0%)) with a specificity of 49.2% (95% CI 43.0% to 55.5%) (see Table 4).

### 3.3. Association of Blood Biomarkers with Outcome

In addition, we evaluated the prognostic information of different logarithmic blood biomarkers, namely, PCT, 25(OH) vitamin D, WBC, urea, and corrected calcium (Table 5). All biomarkers, with the exception of WBC, had a significant association with mortality with AUCs ranging between 0.57 and 0.69. The best prognostic performance was found for PCT (AUC 0.69, 95% CI 0.63 to 0.76) which also significantly improved the GPS to an AUC of 0.74 (95% CI 0.68 to 0.80, *P* < 0.001). Also, corrected calcium had an AUC of 0.69 (95% CI 0.62 to 0.75) and improved the GPS to an AUC of 0.73 (0.67 to 0.79, *P* = 0.002). Urea (AUC 0.57, 95% CI 0.50 to 0.64) showed an AUC of 0.71 (95% CI 0.65 to 0.77) when added to the GPS (*P* = 0.03). Further, 25(OH) vitamin D (AUC 0.61, 95% CI 0.54 to 0.68) tended to improve the GPS to a combined AUC of 0.70 (95% CI 0.64 to 0.77, *P* = 0.053). No significant improvement was found for WBC when adding to the GPS.

We also investigated the overall improvement when combining all markers with the GPS. A combination of PCT, 25(OH) vitamin D, WBC, urea, and corrected calcium without the GPS had an AUC of 0.74 (95% CI 0.68 to 0.81, *P* < 0.001) for outcome prediction. When added to the GPS the overall prognostic accuracy further increased to an AUC of 0.76 (95% CI 0.70 to 0.82, *P* < 0.001).

## 4. Discussion

This cohort study including heterogeneous patients with different types of cancer found a moderate performance of the GPS for early risk assessment and for prediction of 30-day mortality. These results were robust in different subgroups based on age, type of primary origin of the underlying cancer, and presenting symptoms. Further, the GPS was improved by adding different biomarkers, particularly PCT.

An accurate and fast assessment of disease severity and predictions regarding a patient's prospected clinical course assists caregivers and patients with expectations regarding the illness [6]. These assessments and predictions are of particular importance to ensure efficient use of health care and hospital resources and may improve optimal therapeutic options in the initial management of malignancy. This includes decisions regarding site-of-care, diagnostic evaluation, therapeutic measures, and assessment for appropriate early discharge. Interestingly, while for other diseases such as community-acquired pneumonia prediction rules for the ED setting have been proposed and validated (i.e., CURB65, pneumonia severity index) [19], similar ED scores for cancer patients are largely lacking. Herein, our data validating the prognostic performance of the GPS in this patient population is novel and interesting.

The GPS has been widely studied mainly in the medical ward and in surgical patient settings [1–3]. The Glasgow Inflammation Outcome Study included a cohort of 223,303 patients, whereof 22,715 suffered from a cancer. Different routine laboratory markers were found to be associated with mortality in this cohort including CRP and albumin (combined in the mGPS), corrected calcium, alkaline phosphatase, *γ*-glutamyl transferase levels, aspartate transaminase, and alanine transaminase levels (all *P* < 0.001) [20]. Another study compared the outcome of patients with TNM classification of malignant tumours stage II colon cancer undergoing potentially curative surgery and found the mGPS and the fact that patients presented to the ED as an urgency to be independently associated with cancer-specific survival (minimum follow-up: 12 months, median follow-up of survivors: 48 months) [5]. Emergency presentation and mGPS were independently associated with tumour-specific survival in this well-defined population.

Different inflammation-based prognostic scores have been investigated in patients following a cancer diagnosis within two years whereof an elevated mGPS score was associated with a reduced five year overall (AUC 0.718, *P* < 0.001) and cancer specific survival (AUC 0.698, *P* < 0.001) for all investigated cancer types [21]. Our data validates these previous studies and expands the finding to the ED setting and heterogeneous cancer types.

The GPS has later been adapted to the currently more widely used mGPS, based on the finding that an abnormal albumin level alone and cancer-specific survival was similar to that of a normal albumin in patients undergoing potentially curable resection for colorectal cancer [18]. In our cohort, which consists of patients with different types of cancer, the GPS performed better compared to the mGPS which somewhat contradicts these results. However, the latter study excluded patients with clinical evidence for infection and did not involve patients with distant metastases in general (Dukes stage D) and is therefore not comparable to our population.

Kinoshita et al. analysed the performance of the GPS compared to the mGPS and to other scores in patients with hepatocellular carcinoma (HCC) and found on the one hand the GPS to be more suitable as the mGPS and on the other hand an independent association of the GPS with overall survival in the curative treatment group of patients with HCC [22].

Interestingly, in our analysis, the inflammatory and infection marker PCT was found to improve the performance of the GPS. To our knowledge, this is the first study that investigated the prognostic effect of PCT in combination with the GPS. The levels of PCT, a marker for bacterial infections and precursor hormone of calcitonin, have high negative predictive value compared with blood cultures and are therefore useful to rule out sepsis and systemic inflammation in the ED setting [13]. Researchers from the MD Anderson Cancer Center in Texas published several studies about PCT compared to CRP in acute care settings, particularly in the intensive care unit. Shomali and his group found significantly higher PCT levels in septic, nonneutropenic cancer patients and described a significant decrease in PCT values in patients with bacterial infections in response to antibiotics. Interestingly, baseline PCT levels were higher in patients with stage IV disease or metastasis than in those with early stages of cancer [23]. Al Shuaibi investigated PCT levels in febrile patients with hematologic malignancies at the onset of fever and found higher initial PCT levels in patients with definite sepsis and Systemic Inflammatory Response Syndrome (SIRS) compared to those patients without documented infections. However, PCT levels were not significantly different between the SIRS and the sepsis group [24]. And recently, Debiane analysed 114 critically ill adult patients with cancer (51.8% solid cancer, 48.2% haematological cancer) and found an AUC of 0.77 (95% CI 0.67 to 0.87) for PCT testing for 60-day mortality prediction, which was superior compared to CRP testing (AUC 0.62, 95% CI 0.48–0.75) [25]. PCT was also reported to be a prognostic marker in different noncancer conditions such as pneumonia [26] and acute exacerbation of chronic obstructive pulmonary disease (COPD) [27, 28], as well as in other infections [29].

The main strengths of this study are the relatively large population of patients with a cancer-related medical urgency presenting to the ED with follow-up telephone interview information regarding vital status and outcomes assessed on day 30 after ED admission. We had predefined inclusion of consecutive patients with measurement of different blood biomarkers upon admission. Also, patients were heterogeneous in regard to type of cancer and presenting symptoms allowing us to look into different subgroups and assess robustness of our results.

However, the present study has several limitations including single centre design, particular measurement of biomarkers on admission without follow-up values being available, and no detailed information regarding causes of mortality. Our results are therefore more hypothesis generating than definite.

## 5. Conclusion

In conclusion, this study found the GPS to be a moderately helpful prognostic score in patients presenting to the ED with a cancer-related medical urgency, particularly when combined with other biomarkers such as PCT. The benefit of early risk stratification of the GPS in combination with inflammatory biomarkers should be investigated in further trials.

## Figures and Tables

**Table 1:** The Glasgow Prognostic Score (GPS) and the modified Glasgow Prognostic Score (mGPS).

Parameters	Points allocated
The Glasgow Prognostic Score (GPS)	
CRP ≤ 10 mg/L and albumin ≥ 35 g/L	0
CRP > 10 mg/L	1
Albumin < 35 g/L	1
CRP > 10 mg/L and albumin < 35 g/L	2
The modified Glasgow Prognostic Score (mGPS)	
CRP ≤ 10 mg/L and albumin ≥ 35 g/L	0
CRP > 10 mg/L	1
CRP > 10 mg/L and albumin < 35 g/L	2

CRP: C-reactive protein.

**Table 2:** Baseline table.

Characteristics	Overall	Survivors	Nonsurvivors	*P* value
*n* (%)	*341 (100%) *	*260 (76.2%) *	*81 (23.8%) *	
Sociodemographics
Age, years (median, IQR)	68 (60, 75)	68 (58, 75)	69 (61, 75)	*0.20 *
Male gender, *n* (%)	208 (61.0%)	162 (62.3%)	46 (56.8%)	*0.37 *
Diagnosis at ED admission, *n* (%)				
Infection	208 (61.0%)	152 (58.5%)	56 (69.1%)	*0.016 *
Gastrointestinal disease	14 (4.1%)	12 (4.6%)	2 (2.5%)	
Cardiovascular disease	42 (12.3%)	37 (14.2%)	5 (6.2%)	
Worsening of general condition	2 (0.6%)	2 (0.8%)	0 (0.0%)	
Neurological disease	50 (14.7%)	33 (12.7%)	17 (21.0%)	
Other diseases	25 (7.3%)	24 (9.2%)	1 (1.2%)	
Main symptom at ED admission, *n* (%)				
General malaise	60 (17.8%)	40 (15.5%)	20 (25.3%)	*0.46 *
Pain	48 (14.2%)	39 (15.1%)	9 (11.4%)	
Gastrointestinal symptom	84 (24.9%)	65 (25.2%)	19 (24.1%)	
Neurological symptom	44 (13.1%)	36 (14.0%)	8 (10.1%)	
Fever	55 (16.3%)	43 (16.7%)	12 (15.2%)	
Respiratory symptom	46 (13.6%)	35 (13.6%)	11 (13.9%)	
Primary cancer site, *n* (%)				
Solid cancers	276 (80.9%)	207 (75%)	69 (25%)	
Gastrointestinal tract	88 (25.8%)	64 (24.6%)	24 (29.6%)	*0.27 *
Respiratory tract	76 (22.3%)	51 (19.6%)	25 (30.9%)	
Bones, joints, cartilage	6 (1.8%)	6 (2.3%)	0 (0.0%)	
Skin	5 (1.5%)	4 (1.5%)	1 (1.2%)	
Genital, testis, urogenital	58 (17.0%)	46 (17.7%)	12 (14.8%)	
Brain and nervous system	43 (12.6%)	36 (13.8%)	7 (8.6%)	
Haematological cancers	60 (17.6%)	49 (81.7%)	11 (18.3%)	
Hodgkin's lymphoma	4 (1.2%)	4 (1.5%)	0 (0.0%)	
Non-Hodgkin's lymphoma	20 (5.9%)	17 (6.5%)	3 (3.7%)	
Multiple myeloma	13 (3.8%)	12 (4.6%)	1 (1.2%)	
Leukaemia (acute and chronic)	23 (6.7%)	16 (6.2%)	7 (8.6%)	
Not specified or other	5 (1.5%)	4 (1.5%)	1 (1.2%)	
Comorbidities, *n* (%)				
Renal failure	65 (19.1%)	46 (17.7%)	19 (23.5%)	*0.25 *
Diabetes mellitus	46 (13.5%)	36 (13.8%)	10 (12.3%)	*0.73 *
COPD	32 (9.4%)	22 (8.5%)	10 (12.3%)	*0.30 *
Coronary heart disease	20 (5.9%)	16 (6.2%)	4 (4.9%)	*0.68 *
Congestive heart failure	10 (2.9%)	5 (1.9%)	5 (6.2%)	*0.05 *
Blood biomarkers, median (IQR)				
CRP (mg/L)	37.2 (9, 107)	28.35 (7.9, 85.9)	98 (23.2, 161)	*<0.001 *
Albumin (g/L)	31.7 (26.5, 36.6)	33.3 (29, 37.1)	26 (21.6, 31.4)	*<0.001 *
WBC (10^9^/L)	8.3 (5.4, 11.7)	7.9 (5, 11.2)	9.2 (6.3, 13.2)	*0.049 *
PCT (*μ*g/L)	0.14 (0.08, 0.33)	0.13 (0.08, 0.24)	0.28 (0.11, 0.69)	*<0.001 *
Urea (mmol/L)	6.3 (4.7, 9)	6.2 (4.5, 8.7)	6.4 (5.3, 11.8)	*0.062 *
25(OH) vitamin D (nmol/L)	42.2 (24.6, 61.7)	44.9 (27.4, 64)	32.2 (19.9, 47.6)	*<0.001 *
Corr Ca (mmol/L)	2.39 (2.28, 2.51)	2.36 (2.26, 2.46)	2.47 (2.35, 2.66)	*<0.001 *

WBC: white blood cell count; PCT: procalcitonin; 25(OH) vitamin D: 25-hydroxyvitamin D; corr Ca: corrected calcium; COPD: chronic obstructive pulmonary disease.

**Table 3:** Association of GPS and mortality overall and in subgroups.

Population	GPS score	OR (95% CI), *P*	AUC (95% CI)	*P* for heterogeneity
Overall	1 point	1.5 (0.5 to 4.8), *P* = 0.50	0.67 (0.62 to 0.72)	
	2 points	6.4 (2.5 to 16.6), *P* < 0.001		
Age specific				
<60 years	1 point	0.4 (0 to 4.3), *P* = 0.46	0.66 (0.53 to 0.80)	<0.001
	2 points	3.4 (0.9 to 13.6), *P* = 0.08		
≥60 years	1 point	2.8 (0.6 to 14.4), *P* = 0.21	0.66 (0.61 to 0.71)	
	2 points	10.1 (2.3 to 43.3), *P* = 0.002		
Cancer type specific				
All solid cancers	1 point	1.6 (0.5 to 5.2), *P* = 0.48	0.66 (0.61 to 0.72)	0.99
	2 points	5.7 (2.2 to 15.1), *P* < 0.001		
GI tract cancer	1 point	0.6 (0 to 11.5), *P* = 0.77	0.68 (0.59 to 0.76)	
	2 points	6.7 (0.8 to 55.7), *P* = 0.08		
Other than GI tract cancer	1 point	1.8 (0.5 to 6.6), *P* = 0.36	0.67 (0.60 to 0.73)	
	2 points	6.1 (2.1 to 17.9), *P* < 0.001		
Respiratory tract tumour	1 point	1.2 (0.1 to 16.2), *P* = 0.90	0.60 (0.51 to 0.69)	
	2 points	3.8 (0.4 to 33.5), *P* = 0.23		
Diagnosis at ED admission				
Infection	1 point	1.5 (0.4 to 6.2), *P* = 0.59	0.63 (0.57 to 0.70)	0.34
	2 points	4.7 (1.3 to 16.3), *P* = 0.02		
No infection	1 point	0.7 (0.1 to 8.4), *P* = 0.79	0.72 (0.64 to 0.80)	
	2 points	8.4 (1.9 to 38.2), *P* = 0.01		
Cardiovascular	1 point	2.1 (0.2 to 28), *P* = 0.58	0.58 (0.32 to 0.85)	
	2 points	1.9 (0.2 to 15.5), *P* = 0.55		
Main symptom at ED admission				
Worsening of general condition	1 point	3 (0.3 to 35.3), *P* = 0.38	0.61 (0.49 to 0.73)	0.25
	2 points	5.3 (0.6 to 46.8), *P* = 0.13		
Pain	1 point	0.7 (0.1 to 5.4), *P* = 0.72	0.42 (0.22 to 0.62)	
	2 points	0.4 (0.1 to 3.1), *P* = 0.40		
Neurologic symptoms	1 point	1.9 (0.1 to 24.9), *P* = 0.62	0.73 (0.54 to 0.93)	
	2 points	8.2 (1.3 to 52), *P* = 0.03		

GI tract: gastrointestinal tract.

**Table 4:** Diagnostic performance of GPS at different cut-offs.

Cut-point	Sensitivity (CI 95%)	Specificity (CI 95%)	LR+ (CI 95%)	LR− (CI 95%)	PPV (CI 95%)	NPV (CI 95%)
Score 1 point	93.8% (86.2% to 98.0%)	23.8% (18.8% to 29.5%)	1.23 (1.13 to 1.35)	0.26 (0.11 to 0.62)	27.7% (22.5% to 33.4%)	92.5% (83.4% to 97.5%)

Score 2 points	84.0% (74.1% to 91.2%)	49.2% (43.0% to 55.5%)	1.65 (1.42 to 1.93)	0.33 (0.20 to 0.54)	34.0% (27.5% to 41.0%)	90.8% (84.7% to 95.0%)

LR+: positive likelihood ratio; LR−: negative likelihood ratio; PPV: positive predictive value; NPV: negative predictive value.

**Table 5:** Biomarkers alone and in combination with GPS for outcome prediction.

Biomarker	Univariate OR (95% CI), *P*	AUC (95% CI)	Adjusted OR (95% CI), *P*	Combined AUC (95% CI)	*P* value (compared to Score)
PCT	1.6 (1.3 to 1.9), *P* < 0.001	0.69 (0.63 to 0.76)	1.3 (1.1 to 1.6), *P* = 0.007	0.74 (0.68 to 0.80)	<0.001
25(OH) vitamin D	0.5 (0.3 to 0.8), *P* = 0.007	0.61 (0.54 to 0.68)	0.6 (0.4 to 0.9), *P* = 0.022	0.70 (0.64 to 0.77)	0.053
WBC	1.2 (1 to 1.6), *P* = 0.09	0.57 (0.50 to 0.64)	1.2 (1.0 to 1.6), *P* = 0.72	0.69 (0.63 to 0.75)	0.21
Urea	1.6 (1 to 2.4), *P* = 0.033	0.57 (0.50 to 0.64)	1.5 (1.0 to 2.2), *P* = 0.07	0.71 (0.65 to 0.77)	0.03
Calcium	1604 (69 to 37269), *P* < 0.001	0.69 (0.62 to 0.75)	158.8 (6.3 to 4022), *P* = 0.002	0.73 (0.67 to 0.79)	0.002

PCT: procalcitonin; 25(OH) vitamin D: 25-hydroxyvitamin D; WBC: white blood cell count; calcium: corrected calcium.

The blood levels of all biomarkers were log-transformed to achieve normality.

## List of Abbreviations

AUC: Area under the curve
CI: Confidence interval
CRP: C-reactive protein
ED: Emergency department
GPS: Glasgow Prognostic Score
IQR: Interquartile range
mGPS: modified Glasgow Prognostic Score
OR: Odds ratio
PCT: Procalcitonin
ROC curve: Receiver operating characteristic curve
SIRS: Systemic Inflammatory Response Syndrome
25(OH) vitamin D: 25-hydroxyvitamin D
WBC: White blood cell count.
